# Neurocognitive Effects of an Online Brain Health Program and Weekly Telehealth Support Group in Older Adults with Subjective Memory Loss: A Pilot Study

**DOI:** 10.3390/geriatrics9020037

**Published:** 2024-03-14

**Authors:** Ryan M. Glatt, Amylee Amos, David A. Merrill, John F. Hodes, Claudia L. Wong, Karen J. Miller, Prabha Siddarth

**Affiliations:** 1 Pacific Neuroscience Institute and Foundation, Santa Monica, CA 90404, USA; rglatt@pacificneuro.org (R.M.G.); david.merrill@providence.org (D.A.M.); kmiller@pacificneuro.org (K.J.M.); 2 Amos Institute, Seattle, WA 98112, USA; amylee@amosinstitute.com; 3 Department of Psychiatry and Biobehavioral Sciences, David Geffen School of Medicine, University of California Los Angeles, Los Angeles, CA 90095, USA; 4 Providence Saint John’s Cancer Institute, Santa Monica, CA 90404, USA; 5 College of Medicine, Drexel University, Philadelphia, PA 19104, USA; jhodes@pacificneuro.org

**Keywords:** cognitive decline, dementia prevention, aging, health coaching, precision medicine

## Abstract

Introduction: Adopting healthy lifestyle behaviors has the potential to slow cognitive decline in older adults by reducing risks associated with dementia. Curriculum-based group health coaching may aid in establishing behavior change centered for dementia risk factors. Methods: In this pilot clinical care patient group study (*n* = 6), we examined the effects of a six-month online Cognitive Health Program combined with a weekly telehealth support group led by the course creator, and personalized health optimization by a collaborating physician, in older adults with subjective cognitive decline. Cognition was assessed at baseline and post-intervention using a computerized battery. Results: Cognitive changes were estimated with nonparametric tests and effect sizes (Cohen’s d). Results showed significant improvements in global cognition (*p* < 0.03, d = 1.6), spatial planning (*p* < 0.01, d = 2.3), and visuospatial processing (*p* < 0.05, d = 1.1) compared to baseline. Participants reported high levels of satisfaction with the virtual group format and online curriculum. Conclusions: This small pilot study suggests that a virtual six-month personalized health coaching group with self-paced online health education is feasible and potentially efficacious for improving cognition in participants with subjective cognitive complaints. This format may facilitate behavior change to slow cognitive decline. Future studies should include a control group, a larger, more diverse sample as well as assessing mood and other subjective measures.

## 1. Introduction

Brain health is a complex and multidimensional state that encompasses the absence or reduction in brain disease risk factors, as well as preserved cognitive, psychological, and motoric functioning, as verified by measures of brain structure, function, and behavior [1,2]. Research shows that healthy lifestyle behaviors including a Mediterranean-style diet, good sleep, social engagement, physical activity and mental stimulation have a large role to play, not only in enhancing brain health [3], but also in building cognitive reserve [4]. Cognitive reserve refers to the brain’s ability to improvise and find alternate ways of processing and task completion; individuals vary in their ability to process tasks due to distinct reserves that offer protection against brain pathology or age-related cognitive decline. A healthy diet, physical exercise, good sleep quality, stress management and intellectual stimulation all contribute to a more robust cognitive reserve.

Further, incorporation of healthy lifestyle choices may be of particular importance in slowing cognitive decline in older adults with cognitive complaints or impairments [5]. Lifestyle factors, including physical, cognitive and leisure activities, have been shown to have moderately large effects on aspects of cognitive function that contribute to cognitive reserve [6]. The management of vascular risk factors has also been found to reduce the risk of adverse brain health outcomes, including biomarkers related to Alzheimer’s Disease [7,8]. In a large cohort study observing the effects of lifestyle behaviors on brain health outcomes in older adults, over 60% of 4000+ older individuals with higher levels of education who completed a 1-year study follow-up showed improvement in lifestyle domains, particularly those with lower baseline scores. This underscores the potential influence of socio-demographic characteristics, biological, and psychological traits in predicting adherence and success to multidomain lifestyle interventions [9,10].

While research studies have demonstrated that healthy lifestyle behaviors reduce the risk of cognitive decline, the general population, particularly those at risk, falls short in effectively implementing these behaviors. This could be attributed to either a lack of awareness regarding the significance and utility of such lifestyle choices or an inability to adhere to a healthy lifestyle despite recognizing its importance. Health coaching and health education are two different types of intervention that have been utilized to disseminate evidence-based health information. Both offer similar information, but health coaching is specifically geared towards behavior change. Specifically, health coaching is a behavioral approach that empowers individuals to set health-related goals through motivational strategies, conducted under the supervision of a health coach, and includes ancillary support through skillful conversations and behavioral strategies that aim to modify health-related behavior [11]. In contrast, health education, where the primary goal is to provide information about health without personalized interaction strategies like those used in coaching, typically involves didactic teaching techniques or information dissemination alone [12]. Unlike health education, health coaching involves active engagement with clients, and incorporates skillful communication strategies that encourage self-awareness of health-related habits, with support in changing lifestyle behaviors as they relate to an individual’s health goals [13]. These conversations between the coach and the client may encompass various health-related topics, such as dietary patterns, exercise-related behaviors, stress management strategies, sleep hygiene, and other lifestyle behaviors customized according to each person’s specific needs and circumstances.

In a study comparing digital health coaching to digital health education in 216 individuals at risk for Alzheimer’s Disease, it was found that while both health education and health coaching are effective for improving cognition, health coaching may yield increased protection against risk for Alzheimer’s disease by improving cognition or slowing age-related cognitive decline [14]. Recently, the Brain Health Champion study investigated the effects of a health coaching intervention using mobile technology in older adults with mild cognitive impairment or dementia risk factors. The findings revealed that while both health coaching and a counseling/educational group were feasible and led to increased adherence in physical activity and improvements in quality of life, health coaching resulted in greater adherence to the Mediterranean diet, social activities, and cognitive activities [15].

The integration of technology in health coaching and health education can greatly enhance their accessibility and scalability. A meta-analysis of web-based multidomain lifestyle interventions for brain health identified 14 programs designed to optimize brain health that positively influenced outcomes related to brain health and dementia prevention [16]. A literature review of telehealth interventions, which aimed to support health coaching in older adults, found that 10 studies led to improved measures for the following outcomes: hospital admission rates, mortality risk, fatigue levels, HbA1C levels, blood pressure, body weight, physical activity levels, quality of life and user acceptance [17].

Different approaches may be utilized in health coaching, ranging from non-specific, generalized recommendations to personalized recommendations that are customized for each individual based on genetic, behavioral, and biological factors. The latter approach may be more efficacious for the prevention, management, and slowing of neurodegenerative diseases [18] and in the management of age-related cognitive impairment [19]. A pilot study utilizing such a precision medicine approach, known as the Bredesen protocol, in persons with dementia or mild cognitive impairment led to significant improvements in global cognition and symptom severity scores, as well as improvements in aspects of brain volume, as measured by volumetric MRI [20].

Furthermore, it has also been shown that health coaching that includes psychological factors such as self-efficacy, self-care, and stress management may yield greater benefits than individual coaching [21]. Using psychological techniques such as motivational interviewing and cognitive behavioral approaches, coaches can support clients in identifying their sources of motivation, engaging in decisional balance, ‘nudging’ towards health behaviors, providing resources, and helping to initiate behavior change [12]. However, to date, no previous study has examined the effects of combining precision medicine with a group-based health coaching intervention and online health education. Given the already noted benefits of precision medicine in improving cognition in persons with cognitive impairment [20], and the positive effects of health coaching in a similar population [20], we undertook a pilot study combining an online Bredesen protocol-based health coaching intervention with a weekly telehealth support group and biomarker-guided health optimization in older adults with memory concerns. We presently report on the feasibility and cognitive effects of this pilot study.

## 2. Methods

### 2.1. Participants

Patients receiving ongoing care in a multi-disciplinary outpatient brain health clinic (Pacific Brain Health Center, Pacific Neuroscience Institute, Santa Monica, CA, USA) with self-reported cognitive complaints joined a pilot clinical care group. All patients had objectively normal cognition (e.g., Montreal Cognitive Assessment (MoCA) scores of 26 or greater at baseline), were English speaking, had no neurological or major neurocognitive disorders, no uncontrolled moderate–severe mental health conditions, and no limitations on self-reported instrumental activities of daily living; otherwise, no specific exclusionary criteria were set. All six participants were Caucasian individuals, five females and one male, between the ages of 62 and 76 (median = 72) years of age and had 18 years of education (Table 1). All participants had grossly normal MRI scans completed prior to the start of the group as part of standard clinical care to exclude structural or vascular causes of memory loss, along with no evidence of delirium, and normal levels of blood biomarkers related to neurological function (e.g., B12, TSH, RPR). The median MoCA score at the beginning of the intervention was 28/30 (min = 26, max = 30). Five of the six members had APO-E 3/4 genotypes, the remaining group member had an APO-E 3/3, and half the group had a family history of dementia (Table 1).

The data were abstracted from patient records under the Providence Health IRB protocol titled, “Retrospective Review of Patterns of Care in Neurological Disorders” (IRB code JWCI-19-1101, approved 30 April 2022).

### 2.2. Intervention

The pilot study group consisted of six individuals with self-reported cognitive complaints receiving ongoing care at an outpatient brain health center. All six participants were simultaneously enrolled in both a six-month online education program (www.amosinstitute.com, accessed on 17 January 2024) and a parallel telehealth lifestyle support group. The online education program consisted of recorded and written lessons on specific lifestyle topics and allowed participants the flexibility to access and review the content at their convenience, ensuring they could engage with the material in a manner that best suited their schedules. The telehealth intervention was a videoconferencing support group program, designed to complement the online education program, where participants could communicate with their peers and coaches weekly in live online meetings. Thus, the telehealth group sessions served as a forum to discuss topics that were covered in the prior week of the online health education program. During the telehealth sessions, individuals were encouraged to engage in peer coaching, discuss challenges in changing lifestyle behaviors, ask questions, and discuss how to manage stress associated with lifestyle-oriented behavior change utilizing principles of self-efficacy [21]. The telehealth sessions were only live and not recorded. In addition to this six-month intervention, patients had monthly individual clinical (video) appointments with a physician (D.M.) and were given lab-guided personalized health recommendations for dementia risk reduction based on the Bredesen protocol [20,22,23,24] and related approaches [25,26]. As described in these previously published works, this health optimization process included a personalized summary of all lab test results completed during the intervening period, along with medical recommendations on how to improve any suboptimal values. Additionally, these recommendations emphasized a mix of behavioral and lifestyle approaches, including stress management, sleep improvement, exercise, and dietary and nutritional changes; for example, adopting a diet lower in carbohydrates with more whole foods in support of preserving and promoting larger brain volumes.

The registered dietitian (A.A.) who created the online curriculum led the weekly telehealth lifestyle support group, delivered in partnership with the clinic’s doctor (D.M.) and health coach (R.G.), on topics including diet, exercise, sleep, stress, and cognitive training. The online health education platform covered major brain health topics and sub-topics, including nutrition, sleep hygiene, exercise guidelines, stress management, cognitive stimulation, and cognitive training. Sub-topics included aspects of a specific lifestyle domain, such as organic foods, in addition to other dietary topics. Each weekly topic in the online health education platform included a recorded video review of the content, typically approximately 20 min in length, alongside suggested resources (e.g., articles, books, videos, and products) associated with the weekly topic. Topic lessons were released on a weekly basis, with 26 total lessons being available, including an introductory lesson. Weekly question and answer (Q&A) sessions, recorded and posted by A.A. in response to queries from all website subscribers, were also available to group members for viewing.

### 2.3. Outcomes

Cognition was measured at baseline and post-intervention (at 6 months) using a 60 min computerized battery (www.creyos.com, accessed on 17 January 2024, previously known as Cambridge Brain Sciences), which was sent to participants via email along with instructions to complete the assessment in a rested, calm state when they would not be interrupted. The sub-domains of the cognitive assessment included response inhibition, deductive reasoning, spatial planning, verbal reasoning, verbal short-term memory, working memory, episodic memory, attention, spatial short-term memory, mental rotation, visuospatial processing, visuospatial working memory, and global cognition (a composite of all sub-tests).

### 2.4. Statistical Analysis

Demographic variables on participants were summarized using descriptive measures. Changes in cognitive outcome measures from baseline to end of intervention (six months) were examined using the nonparametric Wilcoxon signed-rank test. The primary outcome measure was specified as the global composite score, and sub-test scores were examined in follow-up tests if the primary outcome measure yielded a statistically significant change (*p*-value less than 0.05). In addition, effect sizes (Cohen’s d) are reported for all significant changes.

## 3. Results

All six participants completed the 26 weeks of the online health education platform, and all six had approximately monthly visits with the study physician to optimize their individual health-related biomarkers. The six patients in the weekly telehealth lifestyle support group had a median 90% attendance rate (reasons for absences included travelling and conflicting appointments).

### 3.1. Cognitive Outcome Measures

**Figure 1:**
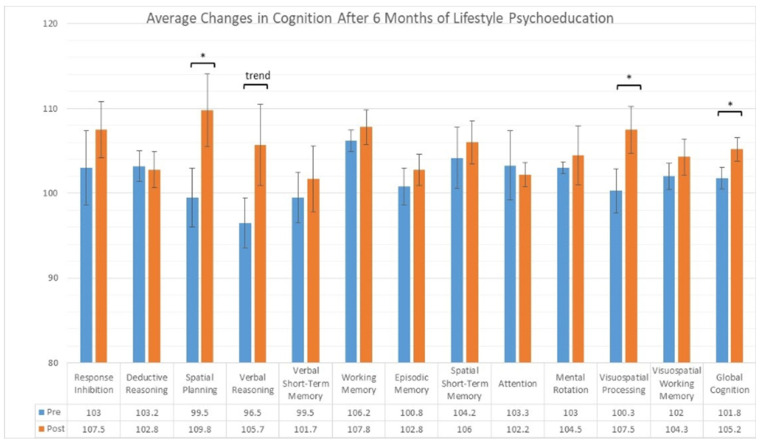
Cognitive domain scores assessed by Cambridge Brain Sciences prior to and after the intervention. * *p* < 0.05; trend *p* < 0.06.

The results of the cognitive tests at baseline compared to six months are shown in Figure 1. Compared to baseline, significant improvement was seen in global cognition (median change = 3.3, IQR = 2.9, *p* < 0.03, d = 1.6). Follow-up tests revealed that spatial planning (median change = 12.0, IQR = 4.0, *p* < 0.01, d = 2.3) and visuospatial processing (median change = 7.0, IQR = 7.0, *p* < 0.05, d = 1.1) improved significantly. Borderline improvement was seen in verbal reasoning (median change = 7.5, IQR = 12.0, *p* < 0.06, d = 1.0). No sub-domain score exhibited significant decline.

### 3.2. Subjective Reports

Subjective measures, while not formally collected through structured surveys, were invited through an open-ended reporting process where participants were encouraged to share their personal experiences with both the telehealth lifestyle support group and the online health education platform after completing the intervention. Participants were provided with a set of guiding questions designed to elicit detailed feedback on aspects such as the perceived effectiveness of the interventions, challenges encountered, areas of improvement, and the overall impact on their health and well-being. These guidelines were intended to ensure comprehensive insights into the participants’ experiences, although they had the freedom to explore any additional topics they deemed relevant. High levels of satisfaction were conveyed with the virtual group format, the online curriculum, and access to the additional online resources. In addition, there was good subjective adherence to multiple lifestyle habits in the areas of sleep, nutrition, and exercise. Some participants reported being confused about what lifestyle practices are concerned with “optimization” versus what aspects of lifestyle behaviors are sufficient for the generalized slowing of cognitive decline.

## 4. Discussion

This small pilot study demonstrated the feasibility of a virtually administered, six-month, weekly health coaching group that addresses multiple lifestyle factors associated with brain health, supplemented by self-paced online health education and physician-assisted health optimization. To our knowledge, this is the first study to combine online, self-paced health education with small-group health coaching in a telehealth context in a clinical setting with a collaborative team of a health coach (R.G.), dietitian (A.A.), and physician (D.M.). This hybrid format of coach–dietitian–doctor–peer led telehealth group with supplementary content is potentially efficacious for improving cognition in patients with subjective cognitive impairment. This innovative intervention led to improvements in global cognition, spatial planning, and visuospatial processing. These results, especially improvements in global cognition, are consistent with prior studies assessing the cognitive benefits of health coaching, including in those with cognitive impairments [14,16,27]. This format may be effective in promoting behavior change that slows cognitive decline and aligns with prior research that incorporates technology into health coaching interventions aimed at improving brain health outcomes [15,16,27].

In this pilot study, we used a computerized cognitive assessment platform (www.creyos.com, accessed on 17 January 2024, previously known as Cambridge Brain Sciences) that was validated and recommended as a potentially supplemental cognitive screening tool in addition to clinical assessments of global cognition, such as the MoCA [28]. This computerized platform generates novel versions of each cognitive task in between trials during the 60 min administration, and attempts to minimize effects from fatigue and practice [29]. The advantages of computerized cognitive testing include automated administration and scoring, and possibly greater sensitivity, validity, and indications of meaningful change relative to individual baselines [30].

In addition to the improvement observed in the objective neurocognitive measures, all participants reported high levels of satisfaction with the virtual group format, the online curriculum, and access to the additional online resources. All participants also reported good subjective adherence to multiple lifestyle habits in the areas of sleep, nutrition, and exercise. Two of the participants reported that they had difficulty understanding which health behaviors to prioritize to address their individual goals.

The incorporation of multiple health care providers and targeted health education was consistent with recommendations from prior research [31], and was perceived as valuable by the participants. The group sessions were generally oriented around topic areas of specific lifestyle domains, rather than guiding group conversations around specific goal-setting with a particular communication style [12], yet this format was highly favored by the participants. A strength of this intervention was the combination of group health coaching combined with digital health education, which may be effective than either intervention separately in terms of adherence [32].

This study is consistent with prior research, such as the Brain Health Champion health coaching intervention, which demonstrated significant and clinically meaningful increases in adherence to social, physical activity, cognitive activity, Mediterranean diet, and quality of life in a clinical setting [33]. The Brain Health Champion intervention consisted of weekly health coaching calls and in-person visits every six weeks, and demonstrated the feasibility of an inexpensive and multi-modal model of health coaching in individuals with dementia, mild cognitive impairment, and subjective cognitive decline [34]. Although the model presented in our study may not be as cost efficient, the online health education platform included in our intervention may be more scalable. A recently published study [35] examined a tablet-based intervention designed for older adults with mild cognitive impairment for staying healthy and following personalized goals, and found a high degree of acceptance and adherence. It is also worth noting that our study was conducted successfully during the COVID-19 pandemic. Based on the result of this pilot study and previous research, online and mobile health coaching programs are feasible for individuals with cognitive complaints, and may lead to improvements in cognition, with the potential to be implemented into routine medical care [27,36].

The primary limitations of this pilot study include the absence of a control group, a homogenous and small sample that limits the ability to draw conclusions and lack of long-term follow-up after the end of the trial. In addition, incorporating additional outcomes such as mood and subjective cognition, as well as measures assessing lifestyle behavior adherence that can be easily administered in standard clinical settings, would be beneficial. It is worth noting that the participants in this study were all Caucasian, of higher socio-economic status, had prior exposure to lifestyle information, experienced prior lifestyle guidance and coaching to varying degrees, were already supervised in a clinical setting by multiple providers, and were highly motivated to adhere and incorporate health behaviors. Thus, the results from this pilot study need to be replicated in a randomized controlled trial with a larger, more diverse population before we can generalize the findings to the broader population of older adults with memory complaints. The health education content could have been better prioritized to address the specific needs and goals of the participants more specifically. This pilot intervention did not employ a particular coaching methodology, such as setting measurable goals, utilizing goal-attainment scales, or employing motivational interviewing to measure successful behavior change. It is of note that the primary interventionist (A.A) was paid to provide the intervention, and owns the online curriculum utilized in this pilot study. To reduce risk of bias, statistical analysis and outcome assessments were conducted independently of the interventionist (A.A). It is also possible that of the 3 of the 13 cognitive outcomes that were improved, the analysis could have considered practice effects and improvement by chance.

Future studies may also seek to incorporate different group health coaching models that are more peer-led, affordable, and involve medical support staff that are more common in various medical practices (e.g., medical assistants, nurse practitioners) [33,34]. Additionally, comparisons between live coaching sessions and purely self-paced curriculum [16,17] could provide valuable insights. Future health coaching interventions should consider incorporating aspects of precision medicine and disease or biomarker-specific interventions and lifestyle behaviors [18,19,20,21,22,23]. It would also be worthwhile to investigate the effectiveness of this hybrid approach with a more automated method of delivering lifestyle information and health coaching services, while conducting more detailed qualitative interviews to better understand the experience of the participants. A further improvement could be to implement an initial co-design phase, which could seek feedback from the target participants and utilize the recommendations in the second phase of the intervention [37]. This would help increase the scalability and enhance the usability and acceptability of such interventions and their potential impact on diverse populations.

## 5. Conclusions

This small pilot study suggests that a clinical intervention combining a self-paced online health education platform with a weekly telehealth lifestyle support group in the context of biomarker-based health assessment and optimization is feasible and potentially efficacious in improving cognition over time. This innovative approach provides a more personalized and comprehensive intervention to promote brain health and cognitive well-being in older adults. By combining the support group and online health education platform, this intervention offers digital accessibility, potentially reducing the cost and accessibility issues associated with lifestyle education and coaching for brain health in persons with subjective cognitive impairments and concerns. While the long-term effects of this intervention and its efficacy in improving cognition remain to be established, our results suggest that these types of lifestyle interventions can have beneficial effects in individuals with subjective cognitive decline and warrant further investigation.

## Figures and Tables

**Table 1:** Demographic and clinical characteristics *.

Age (years)	72 (62–76)
Biological Sex	5 Female, 1 Male
Years of Education	18 (15–20)
MoCA ^$^	28 (26–30)
BMI ^&^	21 (18–27)
APOE-4 risk	3/4 (*n* = 5), 3/3 (*n* = 1)
Family history of dementia	Yes (*n* = 3), No (*n* = 3)
Amyloid status	Positive (*n* = 1), Negative (*n* = 5)

* Numbers indicate median (min–max) for continuous measures; ^$^ Montreal Cognitive Assessment; ^&^ Body Mass Index.

## Data Availability

The data presented in this study are available on request from the corresponding author. The data are not publicly available due to privacy restrictions.
